# Association between BRAF V600E Mutation and Ultrasound Features in Papillary Thyroid Carcinoma Patients with and without Hashimoto’s Thyroiditis

**DOI:** 10.1038/s41598-017-05153-y

**Published:** 2017-07-07

**Authors:** Qin Zhang, Bo-Ji Liu, Wei-Wei Ren, Ya-Ping He, Xiao-Long Li, Chong-Ke Zhao, Yi-Feng Zhang, Wen-Wen Yue, Jia-Yi Zheng, Hui-Xiong Xu

**Affiliations:** 1 Department of Medical Ultrasound, Shanghai Tenth People’s Hospital, Nanjing Medical University, Shanghai, 200072 China; 2 Department of Medical Ultrasound, Shanghai Tenth People’s Hospital, Ultrasound Research and Education Institute, Tongji University School of Medicine, Shanghai, 200072 China; 3 Thyroid Institute, Tongji University School of Medicine, Shanghai, 200072 China; 4 Shanghai Center for Thyroid Diseases, Shanghai, 200072 China; 5 Department of Medical Ultrasound, Huai’an First People’s Hospital, Nanjing Medical University, Huai’an, Jiangsu 223300 China; 6 Department of Pathology, Shanghai Tenth People’s Hospital, Tongji University School of Medicine, Shanghai, 200072 China

## Abstract

To assess the association between BRAF V600E mutation and ultrasound (US) features in papillary thyroid carcinoma (PTC) patients with and without Hashimoto’s thyroiditis (HT). We retrospectively reviewed the US features and status of BRAF V600E mutation in 438 consecutive patients with surgically confirmed PTCs. The association between BRAF mutation and US features were analyzed. In addition, we conducted subgroup analyses in terms of coexistent HT. The BRAF mutation was found in 86.5% of patients (379 of 438). Patient age (OR: 1.028, P = 0.010), age ≥ 50 y (OR: 1.904, P = 0.030), and microcalcification (OR: 2.262, P = 0.015) on US were significantly associated with BRAF mutation in PTC patients. Solid component (OR: 5.739, P = 0.019) on US was the significant predictor for BRAF mutation in patients with HT, while age (OR: 1.036, P = 0.017) and microcalcification (OR: 3.093, P = 0.017) were significantly associated with BRAF mutation in patients without HT. In conclusion, older age and microcalcification are risk factors for BRAF mutation in PTC patients, especially in those without HT. For those with HT, however, PTCs with BRAF mutation tend to be solid on ultrasound. These factors might be considered when making treatment planning or prognosis evaluation.

## Introduction

Papillary thyroid carcinoma (PTC) is the most common subtype of thyroid cancer, which is increasing rapidly worldwide^1^. The rising incidence of PTC is almost entirely attributed to the rapid development of various auxiliary diagnostic technologies^2, 3^.

BRAF V600E mutation (hereafter referred to as the BRAF mutation) is a representative genetic alteration in PTC, leading to abnormal activation of the MAPK pathway, which plays a crucial role in the initiation and progression of PTC^4^. In recent years, this mutation has showed a potential to predict PTC prognosis and can be detected in aspiration specimens preoperatively^5, 6^. Many studies have showed that the occurrence of the BRAF mutation in PTC is correlated with aggressive clinicopathologic features and poor prognosis, such as extrathyroidal invasion, lymph node metastasis, and advanced TNM stage^7–11^. However, other studies have failed to demonstrate the association between BRAF mutation and aggressive clinicopathological characteristics of PTC^12–16^. Although some conflicts exist regarding the relationship between BRAF mutation and clinicopathological characteristics of PTC, BRAF mutation has been reported to be a risk factor for local recurrence and cancer-specific mortality in several studies^17–19^. Besides that, the BRAF mutation plays a role in the lack of avidity of PTC for radioactive iodine^20, 21^. Targeted therapies including a BRAF inhibitor is currently available for iodine-refractory PTC^22, 23^. These above suggest that preoperative understanding of the BRAF V600E mutation status may be helpful for the surgeons and endocrinologists to plan a rational surgical scheme and medical management.

Hashimoto’s thyroiditis (HT) is an autoimmune disease, mostly seen in female. Many authors have reported that the risk of developing PTC increases in those with preexisting HT^24^. On the other side, some investigators have reported that PTC with coexistent HT is associated with lower extrathyroidal invasion, advanced stage, lymph node metastasis and recurrence^25, 26^. Furthermore, there was a study that suggested that concurrent HT could be a shield against PTC progression, even in BRAF-positive patients^27^. Thus, concurrent HT may confuse surgeons and endocrinologists in the decision-making of diagnosis and treatment for PTC.

Diagnostic ultrasound (US) is an important modality for screening of PTC. Recently, several studies have reported the relationship between BRAF mutation and US features in PTC, and the results are inconsistent^28–30^. We hypothesized that US features might be predictive of the status of BRAF mutation in PTC patients and there might be difference in those with HT or not. To confirm these hypotheses, in this study, we analyzed the prevalence of the BRAF mutation in a cohort of PTC patients and aimed to evaluate the association between US features and BRAF mutation in PTC patients with HT or not.

## Materials and Methods

This retrospective study was approved by the Ethical Committee of the Shanghai Tenth People’s Hospital of Tongji University School of Medicine and informed consent was waived for review of patients’ images and records. However, written informed consent was obtained from all patients for BRAF V600E mutation analysis before surgery as a routine procedure. The study was performed in accordance with relevant guidelines and regulations.

### Study population

The medical records of a total of 772 consecutive patients with malignant thyroid tumors were reviewed, who had undergone total or near-total thyroidectomy at the university hospital from December 2015 to October 2016. Only data for those who had both preoperative US findings and postoperative BRAF mutation reports were included. Of them, 202 patients were excluded because of no gene detection records. Another 126 patients were excluded due to US image absence or incompleteness. For the remaining 444 patients, patients were enrolled according to the following inclusion criteria: (a) thyroid tumors were visible on conventional US. (b) US examination within 30 days before surgery. (c) pathological confirmation of thyroid malignancy after surgery. Subsequently, 6 patients were excluded due to other thyroid carcinomas, including 3 poorly differentiated squamous cell carcinomas, 2 follicular carcinomas and 1 medullary carcinoma. Finally, 438 patients with PTCs were included for analysis in this study.

### Conventional US and image analysis

All imaging of the thyroid glands and neck were performed using one of the following high-end US devices: SuperSonic (SuperSonic Imagine, Aix en Provence, France; 4–15 MHz linear transducer), Siemens S2000 (Siemens Medical Solutions, Mountain View, CA, USA; 4–9 MHz or 6–15 MHz linear transducer), IU22 (Philips Medical Systems, Bothell, WA, USA; 5–12 MHz linear transducer) or Logiq E9 (GE Medical Systems, Milwaukee, WI, USA; 5–9 MHz or 6–15 MHz linear transducer). US examinations were performed by one board-certified radiologist in thyroid imaging. The patients were scanned in supine position with dorsal flexion of the head. The target nodule was placed at the center of the screen and the operator constantly adjusted the image settings until optimal images were obtained. Conventional transverse and longitudinal US images were obtained for each target nodule. The US features were evaluated and recorded.

All preoperative US findings were interpreted independently by two board-certified radiologists in thyroid US, who did not know the patients’ identities and BRAF pathologic results confirmed by surgical specimen. When discordance appeared for the evaluation between the two radiologists, another senior radiologist in thyroid US evaluated the images and made the final decision. If the preoperative US and pathological examination suggested multifocality of PTC, we selected the largest nodule confirmed as PTC. The following conventional US features of the target nodule were assessed: size on US (≤10 mm in largest diameter, >10 mm in largest diameter), location (upper, middle, low, mixed), nodule number (single, multiple), nodule distribution (unilateral, bilateral), capsule contact (no, yes), capsule involvement (no, yes), echogenicity (hypo-, iso- or hyper-echogenicity in comparison with the surrounding thyroid tissue), internal component (solid, cystic-solid), shape (regular, irregular), margin (well-defined, ill-defined), calcification (no calcification, microcalcification ≤1 mm in diameter^31^, macrocalcification >1 mm in diameter with or without acoustic shadow), ratio of height and width (taller than wide, wider than tall), vascularity (absent, slight, rich), blood flow distribution (absent, internal, peripheral, mixed with both internal and peripheral flow).

### BRAF mutation analysis

BRAF mutation analysis was performed at the Pathology Department of the university hospital. DNA was extracted from postoperative tissue samples using the QIAGEN QIAamp DNA FFPE Tissue Kit (56404, QIAGEN) according to the manufacturer’s protocol and in 50 μl of buffer ATE (included in the kit). The absorbance of the extracted DNA was measured by a Merinton SMA4000 spectrophotometer (Merinton Inc., Beijing, China). The DNA was diluted with distilled water to a concentration of approximately 2–3 ng/μl. We tested the BRAF mutation using a human BRAF V600E ARMS-PCR kit (Amoy Diagnostics Co. Ltd, Xiamen, China) approved by the China Food and Drug Administration (CFDA). The quality of the extracted DNA was verified by the amplification of a housekeeping gene reported in the HEX channel. Amplification was performed using the following cycling conditions on an ABI Prism 7500 thermocycler (Life Technologies, Carlsbad, California, USA): 95 °C for 5 min; 15 cycles of 95 °C for 25 s, 64 °C for 20 s, 72 °C for 20 s; and 31 cycles of 93 °C for 25 s, 60 °C for 35 s, 72 °C for 20 s. The FAM and HEX signals were collected at 60 °C. At last, the run files were analyzed and interpreted in accordance with the manufacturer’s instructions specified.

### Statistical analysis

Statistical analyses were performed using the SPSS software (version 20.0, Chicago, IL, USA). Continuous variables were presented as mean ± SD and categorical variables were presented as the number of cases, with percentage (%). Continuous variables were compared by independent two-sample t test. Chi-square test or Fisher’s exact test was used to analyze the categorical variables as appropriate. Univariate analysis was performed to analyze the correlation between the predicting factors and BRAF mutation. Odds ratios (ORs) with 95% confidence intervals (CIs) were calculated. Sub-analysis was performed according to the status of coexisting HT or not. P value of less than 0.05 was considered statistically significant.

## Results

**Figure 1:**
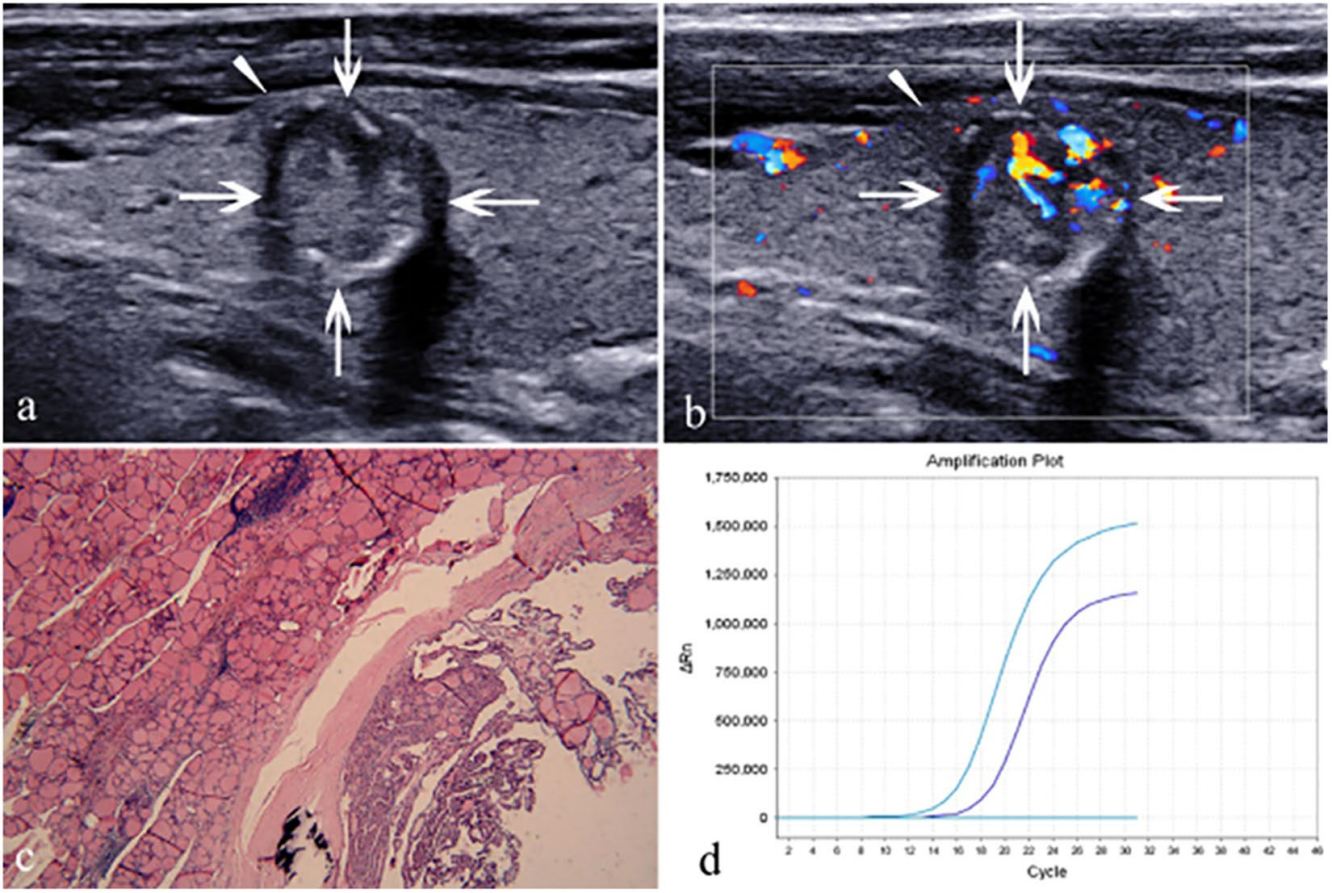
A 54-year-old woman with papillary thyroid carcinoma. (**a**) A solid, hypoechogenic, irregular and ill-defined nodule (arrows) with capsule contact and involvement (triangle) is shown on US and macrocalcifications with acoustic shadow are found in the nodule. (**b**) The nodule shows rich blood flow distributed internally and peripherally on color Doppler US. (**c**) Pathological examination confirms the diagnosis of papillary thyroid carcinoma with local invasion of thyroid capsule and Hashimoto’s thyroiditis. (haematoxylin-eosin stain; original magnification, ×40). (**d**) The amplification plot of BRAF shows BRAF mutation type. The purple curve, representative of the BRAF sample, is up between the two light blue reference lines. The horizontal light blue line suggests BRAF-negative, while the upward light blue curve suggests BRAF-positive.

**Figure 2:**
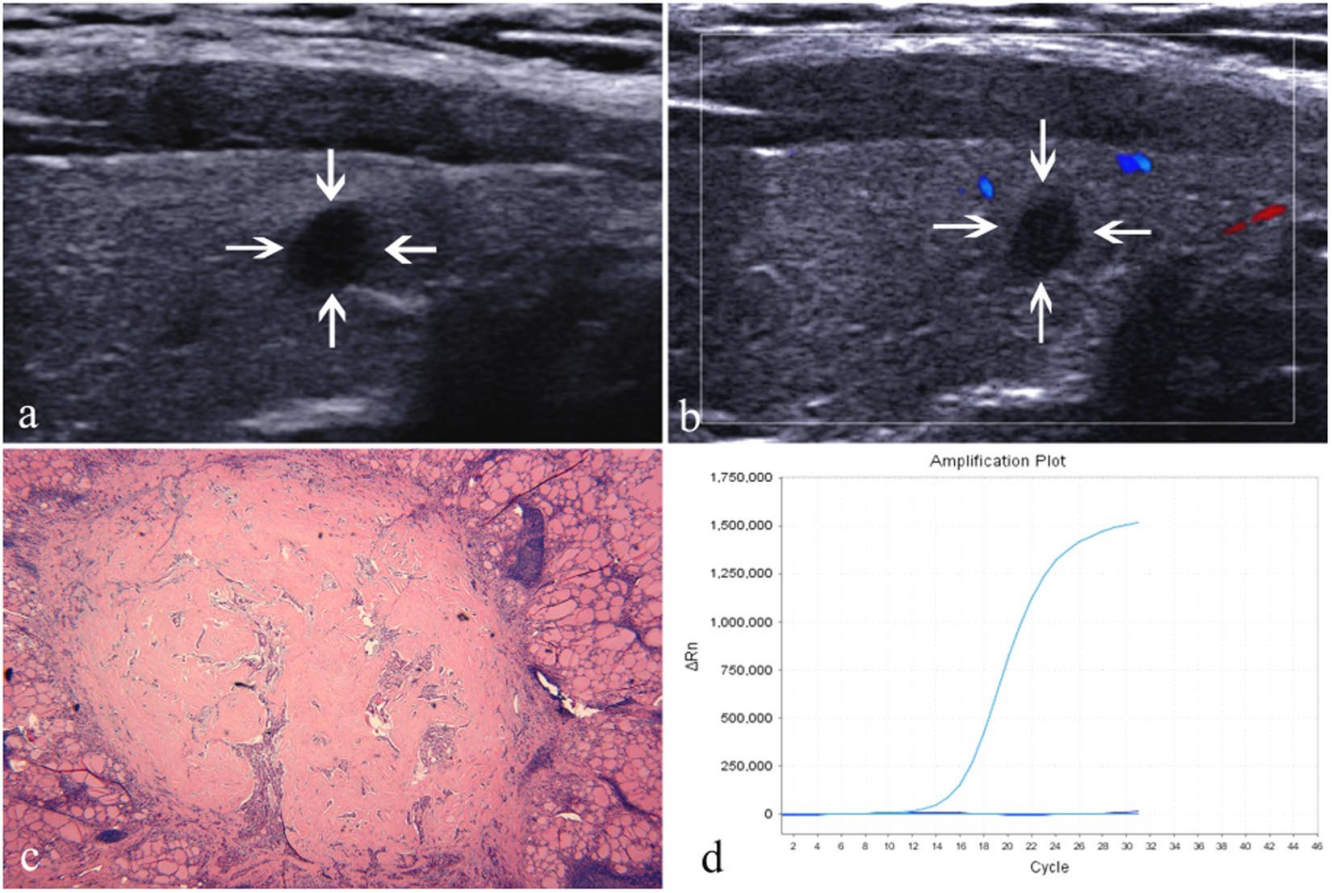
A 39-year-old woman with papillary thyroid microcarcinoma. (**a**) A solid, hypoechogenic, regular, well-defined and taller-than-wide nodule (arrows) without capsule contact is shown on US and no calcification is found in the nodule. (**b**) No blood flow is shown on color Doppler US. (**c**) Pathological examination confirms the diagnosis of papillary thyroid microcarcinoma and Hashimoto’s thyroiditis. (haematoxylin-eosin stain; original magnification, ×40). (**d**) The amplification plot of BRAF shows BRAF wild type. The purple curve, representative of the BRAF sample, overlaps the horizontal light blue reference line which suggests BRAF-negative.

The subjects included 379 (86.5%) BRAF-positive PTCs (Fig. 1) and 59 (13.5%) BRAF-negative PTCs (Fig. 2). Among these subjects, 163 PTC patients were conformed with HT and 275 without HT by histopathology after surgery. The mean nodule size was 11.69 ± 8.59 mm (range, 3–84 mm).

In all PTC patients, the BRAF-positive patients were significantly older than those without BRAF mutation (47.4 ± 13.8 years Vs. 42.4 ± 12.3 years, P = 0.009). Patients older than 50 years were more prone to be BRAF-positive (P = 0.028). Among the US characteristics, only calcification was significantly different between positive BRAF mutation and negative BRAF mutation (P = 0.045). None of the other factors such as gender, size, location, nodule number, nodule distribution, capsule contact, capsule involvement, echogenicity, internal component, shape, margin, ratio of height and width, vascularity and blood flow distribution were significantly associated with BRAF mutation in PTC patients (all P > 0.05) (Table 1). Univariate analysis demonstrated that the predictors such as age (OR: 1.028, P = 0.01), age ≥ 50 years (OR: 1.904, P = 0.03) and microcalcification (OR: 2.262, P = 0.015) were significantly associated with BRAF mutation in PTC patients (Table 2).

**Table 1:** Characteristics of the patients and US according to the BRAF mutation status.

Characteristics	Total PTCs (n = 438)		P value
	BRAF (+)	BRAF (−)	
	n = 379 (86.5%)	n = 59 (13.5%)	
Age			
Mean ± SD (y)	47.4 ± 13.8 (20–84)	42.4 ± 12.3 (10–67)	0.009
<50/≥50 y	199 (52.5)/180 (47.5)	40 (67.8)/19 (32.2)	0.028
Gender			
Male/female	91 (24)/288 (76)	9 (15.3)/50 (84.7)	0.136
Size on US			
Mean ± SD (mm)	11.8 ± 8.6 (3–84)	11.3 ± 8.4 (3–44)	0.674
≤10/>10 mm	219 (57.8)/160 (42.2)	37 (62.7)/22 (37.3)	0.475
Location			
Upper/middle/low/mixed	40 (37.2)/83 (21.9)/27 (7.1)/229 (60.4)	3 (5.1)/15 (25.4)/3 (5.1)/38 (64.4)	0.516
Nodule number			
Single/multiple	136 (35.9)/243 (64.1)	18 (30.5)/41 (69.5)	0.421
Nodule distribution			
Unilateral/bilateral	173 (45.6)/206 (54.4)	27 (45.8)/32 (54.2)	0.987
Capsule contact			
Yes/no	128 (33.8)/251 (66.2)	16 (27.1)/43 (72.9)	0.311
Capsule involvement			
Yes/no	27 (7.1)/352 (92.9)	5 (8.5)/54 (91.5)	0.919
Echogenicity			
Hypo-/iso- or hypergenicity	357 (94.2)/22 (5.8)	53 (89.8)/6 (10.2)	0.323
Internal component			
Solid/cystic-solid	363 (95.8)/16 (4.2)	53 (89.8)/6 (10.2)	0.104
Shape			
Regular/irregular	154 (40.6)/225 (59.4)	19 (32.2)/40 (67.8)	0.218
Margin			
Ill-defined/well-defined	164 (43.3)/215 (56.7)	28 (47.5)/31 (52.5)	0.547
Calcification			
Micro-/macro-/no calcification	210 (55.4)/22 (5.8)/147 (38.8)	42 (71.2)/4 (6.8)/13 (22)	0.045
Ratio of height and width			
Taller than wide/wider than tall	105 (27.7)/274 (72.3)	18 (30.5)/41 (69.5)	0.656
Vascularity			
Absent/slight/rich	135 (35.6)/231 (60.9)/13 (3.4)	21 (35.6)/37 (62.7)/1 (1.7)	0.776
Blood flow distribution			
Absent/internal/peripheral/mixed	135 (35.6)/138 (36.4)/24 (6.3)/82 (21.6)	21 (35.6)/22 (37.3)/4 (6.8)/12 (20.3)	0.996

^#^Numbers in parentheses are percentages or ranges.

US: ultrasound, PTC: papillary thyroid carcinoma.

**Table 2:** Univariate analyses of factors for predicting BRAF mutation.

Characteristics	β Coefficient	Odds Ratios	95% Confidence Interval	P value
Total PTC				
Age	0.028	1.028	1.007–1.050	0.010
Age ≥50 y	0.644	1.904	1.064–3.408	0.030
Microcalcification	0.816	2.262	1.173–4.362	0.015
PTC with HT				
Solid component	1.747	5.739	1.340–24.588	0.019
PTC without HT				
Age	0.035	1.036	1.006–1.066	0.017
Microcalcification	1.129	3.093	1.222–7.829	0.017

PTC: papillary thyroid carcinoma, HT: Hashimoto’s thyroiditis.

In PTC patients with HT, BRAF mutation was found in 136 (83.4%) patients. It was statistically different only for internal component on US between those with BRAF mutation and those without (P = 0.034). Patient age, gender and other US features had no associations with BRAF mutation status (all P > 0.05). On the other hand, in PTC patients without HT, BRAF mutation was found in 243 (88.4%) patients. The BRAF-positive patients were significantly older than those without BRAF mutation (47.5 ± 13.5 years Vs 41.3 ± 13.2 years, P = 0.015) and calcification (P = 0.038) was found to be significantly different. There were no significant differences in gender and other US features (all P > 0.05) (Table 3). Univariate analysis suggested that solid component (OR: 5.739, P = 0.019) was the significant predictor for BRAF mutation in PTC patients with HT. Meanwhile, age (OR: 1.036, P = 0.017) and microcalcification (OR: 3.093, P = 0.017) were significantly associated with BRAF mutation in those without HT (Table 2).

**Table 3:** Characteristics of the patients and US according to the BRAF mutation and concurrent HT status.

Characteristics	Total PTCs (n = 438)					
	With HT, n = 163 (37.2%)		P value	Without HT, n = 275 (62.8%)		P value
	BRAF (+)	BRAF (−)		BRAF (+)	BRAF (−)	
	n = 136 (83.4%)	n = 27 (16.6%)		n = 243 (88.4%)	n = 32 (11.6%)	
Age						
Mean ± SD (y)	47.1 ± 14.2 (20–84)	43.7 ± 11.2 (23–65)	0.171	47.5 ± 13.5 (20–80)	41.3 ± 13.2 (10–67)	0.015
<50/≥50 y	72 (52.9)/64 (47.1)	18 (66.7)/9 (33.3)	0.190	127 (52.3)/116(47.7)	22 (68.8)/10 (31.2)	0.078
Gender						
Male/female	23 (16.9)/113 (83.1)	4 (14.8)/23 (85.2)	1.000	68 (28)/175 (72)	5 (15.6)/27 (84.4)	0.137
Size on US						
Mean ± SD (mm)	11.6 ± 5.9 (5–33)	12.4 ± 9.4 (4–44)	0.670	11.8 ± 9.8 (3–84)	10.3 ± 7.4 (3–35)	0.382
≤10/>10 mm	71 (52.2)/65 (47.8)	14 (51.9)/13 (48.1)	0.973	148 (60.9)/95 (39.1)	23 (71.9)/9 (28.1)	0.229
Location						
Upper/middle/low/mixed	14 (10.3)/32 (23.5)/12 (8.8)/78 (57.4)	2 (7.4)/7 (25.9)/1(3.7)/17 (63)	0.775	26 (10.7)/51 (21)/15 (6.2)/151 (62.1)	1 (3.1)/8 (25)/2 (6.2)/21 (65.6)	0.589
Nodule number						
Single/multiple	51 (37.5)/85 (62.5)	9 (33.3)/18 (66.7)	0.682	85 (35)/158 (65)	9 (28.1)/23(71.9)	0.442
Nodule distribution						
Unilateral/bilateral	62 (45.6)/74 (54.4)	12 (44.4)/15 (55.6)	0.913	111 (45.7)/132 (54.3)	15 (46.9)/17 (53.1)	0.898
Capsule contact						
Yes/no	40 (29.4)/96 (70.6)	9 (33.3)/18 (66.7)	0.685	88 (36.2)/155 (63.8)	7 (21.9)/25 (78.1)	0.109
Capsule involvement						
Yes/no	9 (6.6)/127 (93.4)	4 (14.8)/23 (85.2)	0.295	18 (7.4)/225 (92.6)	1(3.1)/31 (96.9)	0.598
Echogenicity						
Hypo-/iso- or hypergenicity	357 (94.2)/22 (5.8)	53(89.8)/6 (10.2)	0.323	357 (94.2)/22 (5.8)	53 (89.8)/6 (10.2)	0.323
Internal component						
Solid/cystic-solid	132 (97.1)/4 (2.9)	23 (85.2)/4 (14.8)	0.034	231 (95.1)/12 (4.9)	30 (93.8)/2 (6.2)	1.000
Shape						
Regular/irregular	44 (32.4)/92 (67.6)	9 (33.3)/18 (66.7)	0.921	110 (45.3)/133 (54.7)	10 (31.2)/22 (68.8)	0.133
Margin						
Ill-defined/well-defined	67 (49.3)/69 (50.7)	12 (44.4)/15 (55.6)	0.647	97 (39.9)/146 (60.1)	16 (50)/16 (50)	0.276
Calcification						
Micro-/macro-/no calcification	78 (57.4)/9 (6.6)/49 (36)	17 (63)/3 (11.1)/7 (25.9)	0.494	132 (54.3)/13 (5.3)/98 (40.3)	42 (71.2)/4 (6.8)/13 (22)	0.038
Ratio of height and width						
Taller than wide/wider than tall	36 (26.5)/100 (73.5)	6 (22.2)/21 (77.8)	0.645	69 (28.4)/174 (71.6)	12 (37.5)/20 (62.5)	0.288
Vascularity						
Absent/slight/rich	53 (39)/78 (57.4)/5 (3.7)	8(29.6)/18 (66.7)/1 (3.7)	0.651	82 (33.7)/153 (63)/8 (3.3)	13 (40.6)/19 (59.4)/0 (0)	0.473
Blood flow distribution						
Absent/internal/peripheral/mixed	53 (39)/49 (36)/10 (7.4)/24 (17.6)	8 (29.6)/10 (37)/2 (7.4)/7 (25.9)	0.719	82 (33.7)/89 (36.6)/14 (5.8)/58 (23.9)	13 (40.6)/12 (37.5)/2 (6.2)/5 (15.6)	0.743

^#^Numbers in parentheses are percentages or ranges.

US: ultrasound, HT: Hashimoto’s thyroiditis, PTC: papillary thyroid carcinoma.

## Discussion

The frequency of BRAF mutation in PTC patients was 86.5% (379/438) in this series, which is higher than that in other reports from mainland China^6, 32–34^. The incidence of BRAF V600E mutation in PTC also varies from 25% to 90% in other countries ^6, 13, 35, 36^. The highest prevalence of the BRAF mutation was up to 90% in Korean PTC patients^35^. These differences in prevalence might result from differences in subjects’ geographical regions^37^ and research methodology. Some studies indicated that iodine supplementation programs in China may have resulted in the observed high prevalence of BRAF mutations^38^. In this study, the BRAF mutations were tested from the postoperative tissue samples using the ARMS-PCR method, which permits the identification of mutant alleles with a penetration of 1% in a specimen^39, 40^. In previous reports, the limit of detection for mutant alleles is usually as high as 10% for the Sanger sequencing method, by which low-frequency BRAF mutations might have not been identified. Additionally, specimens collected from the fine-needle aspiration (FNA) nodules of PTC might not be large enough to identify all BRAF changes. Thus, the proper value of the quantification of the mutated allele remains in argument and requires further research. On the other hand, we found that the incidence of BRAF mutation was less frequently in Chinese PTC patients with HT than those without HT (83.4% vs. 88.4%), although the difference was not statistically significant. This result is consistent with two previous studies in a Korean population (64% vs. 90% and 72.9% vs. 95.3% respectively)^41, 42^.

Recently, several studies were performed to evaluate the relationship between BRAF mutation status and US features in PTC, and those reports presented inconsistent results^28–30^. A large-scale Korean study on patients with PTC reported no associations between BRAF V600E mutation and US features including composition, echogenicity, margin, calcification or shape, regardless of tumor size^43^. Another Korean study on 339 patients with PTMC also reported no significant difference except tumor size^29^. Conversely, a recent study on 115 Italian with PTC larger than 10 mm found that BRAF-positivity was associated with most suspicious US findings, including taller-than-wide shape, ill-defined margins, hypoechogenicity, micro/macrocalcifications, and absent halo but was not associated with non-cystic composition^44^. Moon *et al*. revealed that only an irregular shape was found to have a negative association with BRAF V600E status^30^. Hwang *et al*. also found a significant association of BRAF mutation with a lower rate of calcifcations detected on US^28^. Our result suggests that older age (especially than 50 years) had significant association with BRAF mutation in Chinese PTC patients, similar to the previous Chinese study^34^. Microcalcification was the only US feature associated with BRAF mutation status. Gender and other US characteristics such as size, location, nodule number, nodule distribution, capsule contact, capsule involvement, echogenicity, internal component, shape, margin, ratio of height and width, vascularity and blood flow distribution were not related to BRAF mutation. Our results of total PTC patients support those of previous reports, which propose that most US features are not related to BRAF mutation. The variations between ours and the above mentioned studies might result from different study population and DNA methodology.

Until present, there has been no report about the association between US features and BRAF mutation in PTC patients according to the status of concurrent HT. In this study, the results from the patients without HT were similar to those from the total PTCs, which showed older age and microcalcification were the high risk factors for BRAF mutation. Interestingly, the PTC patients with HT exhibited a different presentation. Only solid component on US was associated with BRAF mutation. This phenomenon may be explained with that the solid component of some BRAF-negative nodules might be derived from dense fibrosis keloid-like bands which subvert the normal thyroid structure and change the gland to a lobular appearance^45^. Hence, solid component alone was identified as a predictor for BRAF mutation PTC in HT patients, which indicates that another perspective is in need for HT patients.

Our study has two strengths. Firstly, this is a large single-center study on PTC and BRAF mutation, which limits heterogeneity of population. Secondly, we analyzed the association of BRAF mutation with various US features in PTC patients with and without concurrent HT. It provides additional information for the management of clinical BRAF V600E detection. However, several limitations also existed in this study. Firstly, a selection bias might be present that only patients with PTC confirmed by postoperative pathology included in this retrospective study. The US features of PTC patients who did not undergo surgery but had BRAF mutations were not taken into account. Secondly, this study included all types of PTCs, such as conventional PTC and variants. Tall-cell PTC is well established as a variant with more aggressive behaviors, such as extrathyroidal invasion, lymph node metastasis and recurrence^46^. The variants might play a role in the contribution to the statistical analysis in the present study. Unfortunately, the number of the variants was small in the current study so that a subgroup analysis was not achievable. Further study with sufficient PTC variants might be helpful to evaluate the association between US features and PTC variants. Finally, we just evaluated the associations of conventional US characteristics with the gene of BRAF V600E. In future, US elastographic evaluation can be conducted to other genes related to PTC, such as TERT promoter mutation.

## Conclusion

In conclusion, our study demonstrated a high prevalence of BRAF V600E mutation in PTC patients. Older age and microcalcification are high risk factors for BRAF mutation in PTC patients, especially in those without HT. However, those with concurrent HT show an alien correlation that tumor with BRAF mutation tends to be solid on US. This confounding factor of concurrent HT should be considered in the prediction of the status of BRAF mutation in PTC patients.
